# Cytotoxic T-lymphocyte-associated 4 protein expression is associated with a high international prognostic score in advanced-stage classical Hodgkin lymphoma

**DOI:** 10.1186/s13104-024-06853-1

**Published:** 2024-07-08

**Authors:** Flora Dameria Pangaribuan, Maria Francisca Ham, Mutiah Mutmainnah, Agnes Stephanie Harahap

**Affiliations:** 1 Anatomical Pathology Department, Persahabatan Hospital, Jakarta, 13230 Indonesia; 2 Anatomical Pathology Department, Faculty of Medicine Universitas Indonesia, Dr. Cipto Mangunkusumo National Central General Hospital, Jakarta, 10430 Indonesia; 3 Faculty of Medicine Universitas Indonesia, Human Cancer Research Center-Indonesian Medical Education and Research Institute, Jakarta, 10430 Indonesia; 4 Faculty of Medicine, Universitas Muhammadiyah Palembang, Palembang, 30263 Indonesia

**Keywords:** Advanced-stage, Cytotoxic T-lymphocyte-associated protein 4, Hodgkin lymphoma, International prognostic score

## Abstract

**Objective:**

Twenty percent of all classical Hodgkin lymphoma (CHL) cases relapse and recur, especially in advanced stages with a high International Prognostic Score (IPS). Cytotoxic T-lymphocyte-associated protein 4 (CTLA-4) is a regulatory molecule that can inhibit the immune response and is related to tumor aggressiveness. This study aimed to determine the relationship between CTLA-4 expression in advanced-stage CHL and IPS, identifying it as a potential therapy target.

**Results:**

In advanced-stage CHL, the group with a high IPS exhibited significantly higher mean CTLA-4 expression compared to the group with a low IPS (p = 0.003).The group with Hb level < 10.5 g/dl, leukocyte count > 15,000/µL, lymphocyte count < 8%, albumin level < 4 g/dl, and stage 4 exhibited higher CTLA-4 expression than the other group, although only leukocyte count and stage showed statistical significance (p = 0.004 and p = 0.020). Mean CTLA-4 expression was 239.84 ± 76.36 for nodular sclerosis, 293.95 ± 147.94 for mixed cellularity, 271.4 ± 23.56 for lymphocyte depleted, and 225.2 for lymphocyte-rich subtypes. The results suggest that CTLA-4 expression is associated with adverse prognostic factors in the IPS for advanced-stage CHL, supporting the notion that immune checkpoints play a role in cancer progression.

## Introduction

Classical Hodgkin lymphoma (CHL) is a lymphoid malignancy with a challenging diagnosis and treatment. Although most CHL tumours show good prognosis, with an 80% recovery rate, some cases present with relapse and usually are diagnosed at advanced stages [1]. The International Prognostic Score (IPS) uses risk factors in an advanced-stage CHL case to determine treatment plans and predict relapse or recurrence. These risk factors include age, sex, stage, haemoglobin (Hb), leukocyte count, lymphocyte count and serum albumin level [2–5]. Advanced-stage CHL with a higher IPS has a higher risk of recurrence or relapse [6]. Advanced-stage CHL with IPS 0–3 shows 83% overall survival and IPS ≥ 4 59% [7].

CHL exhibits characteristic morphological features marked by Reed-Sternberg cells or Hodgkin cells with beneficial enrichment of inflammatory cells such as lymphocytes, eosinophils, histiocytes and other immune cells as the background, constituting the tumour microenvironment (TME) [8, 9]. The TME is constructed by the interaction of tumour cells and normal cells, such as stromal cells, fibroblasts, immune cells, and the extracellular matrix. The tumour cells and TME of CHL produce molecules that induce cell proliferation, survival, and immune escape to promote tumour cell growth [10]. A significant component of the TME in CHL is cytotoxic T-lymphocyte-associated protein 4 (CTLA-4), a regulatory molecule that can inhibit the immune response, therefore decreasing the ability of the immune system to destroy tumour cells and is related to tumour aggressiveness. Expression and interaction of CTLA-4 and the TME are thought to correlate with the stage of CHL, as they can reflect the extent of immune evasion by the tumour [11, 12].

Therapeutic management of CHL continues to develop. One of the new therapies to combat relapse/recurrent CHL is through the immune system regulation pathway and immunophysiological interaction in the TME, such as use of immune checkpoint inhibitors (ICIs). This therapy aims to restore the patient’s natural immune response against tumour cells. CTLA-4 inhibitors are ICIs that have a promising future in advanced-stage CHL therapy [13, 14]. Studies to date regarding the prognosis of malignancy cases treated with CTLA-4 show differing conclusions. The connection between CTLA-4 and the IPS in CHL is part of a broad landscape in immune system interactions. CTLA-4 can influence the behaviour of CHL, which is reflected in prognostic tools such as the IPS. This study will elucidate the immunoexpression profile of CTLA-4 in advanced-stage CHL and its correlation with the IPS.

## Material and methods

### Ethics

This study was approved by The Ethics Committee of Universitas Indonesia, Jakarta, Indonesia, in accordance with the Declaration of Helsinki with protocol number KET-127/UN2.F1/ETIK.PPM.00.02/2022. Informed consent was waived by The Ethics Committee of Universitas Indonesia, Jakarta, Indonesia (letter number ND-884/UN2.F1/ETIK/PPM.00.02/2023).

### Sample collection

This was a retrospective study that was performed at the Anatomical Pathology Department, Faculty of Medicine Universitas Indonesia, Dr. Cipto Mangunkusumo Hospital, Jakarta, Indonesia. A total of 40 advanced-stage CHL cases available on the Anatomical Pathology Department archives from the 1st of January 2013 to the 30th of September 2021 were enrolled by using consecutive sampling techniques. All cases were confirmed by comprehensive immunohistochemistry (IHC) examination. Cotswolds-*modified* Ann Arbor was used as the staging system. Stage 2 with B-symptoms and/or bulky disease, stage 3, and stage 4 are categorized as advanced stage. The IPS is determined by factors such as age, sex, Hb value, leukocyte count, lymphocyte count, albumin value, and clinical stage. The samples were divided into two groups: advanced-stage CHL with low IPS (cases with 0–3 IPS factors) and high IPS (cases with > 4 IPS factors). Patients' clinical data, including age, sex, laboratory findings, imaging results, therapy history, and clinical stage, were taken from medical records.

### CTLA-4 immunohistochemistry examination

Formalin-fixed paraffin-embedded tissue blocks (FFPE) was cut (3–5 µm), placed on a glass slide, and heated above a slide warmer at 56.5–60.0 °C for 60 min. Incubation was performed in Tris–EDTA with a decloaking chamber followed by submerging in peroxidase blocking solution and protein block solution. The slides were then incubated with an anti-CTLA-4 primary antibody (Biocare Medical, LLC. UMAB249 clone, CA, USA) at a 1:150 dilution for 1 h. Postprimary (Rabbit anti mouse IgG (< 10 μg/mL) in 10% (v/v) animal serum) and secondary (Novolink™ Polymer. Anti-rabbit Poly-HRP-IgG (< 25 μg/mL) containing 10% (v/v) animal serum in tris-buffered saline/0.09% ProClinTM 950) antibodies by Novolink detection kit ^®^ RE7140-CE (Leica Biosystems, Newcastle, UK) were then added for 30 min. Negative and positive controls were included with each preparation. Negative controls were performed by omitting the antibody step in each case. The positive control for CTLA-4 was tonsil FFPE and taken from the laboratory archive.

**Fig. 1:**
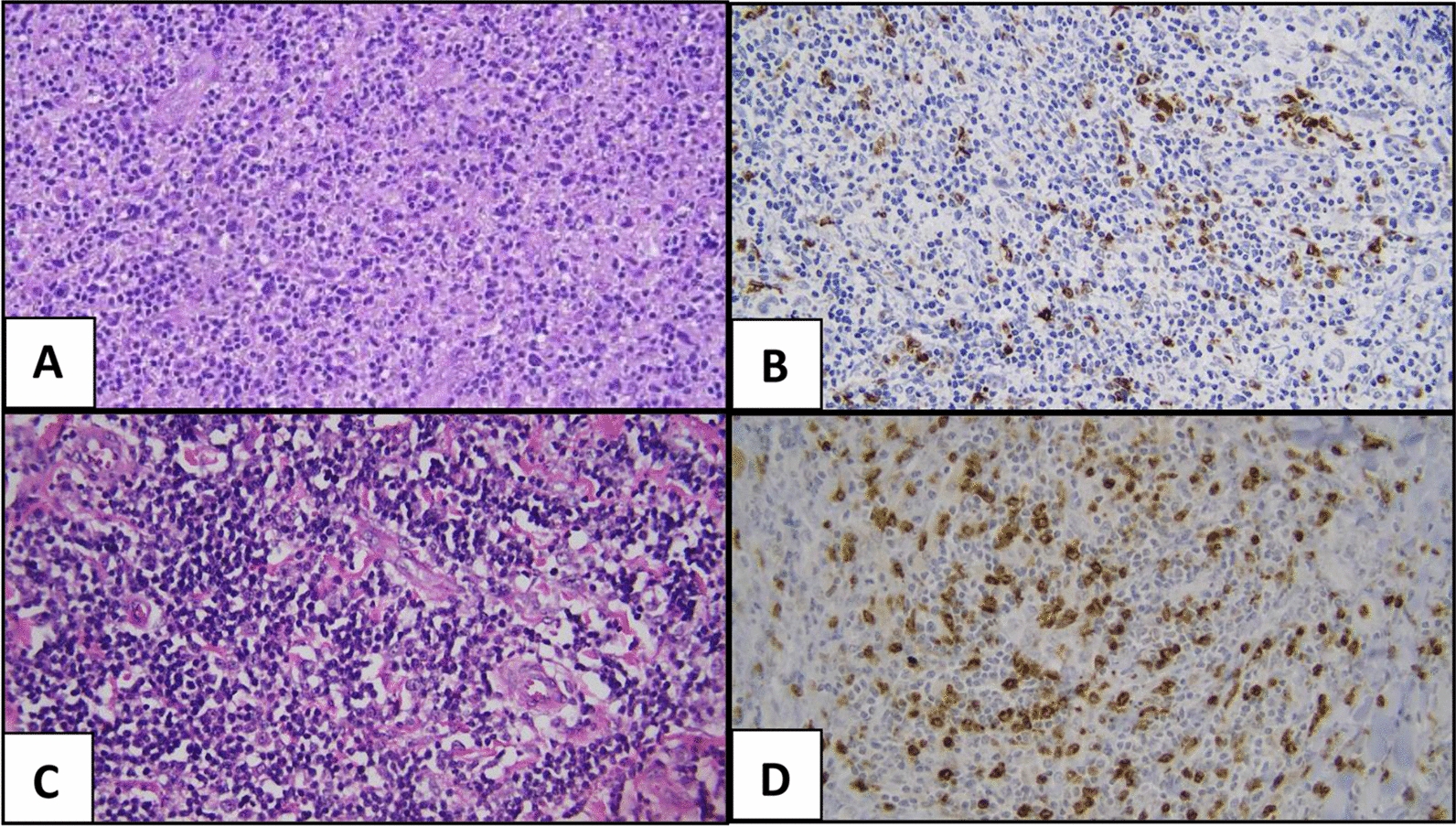
Expression of CTLA-4 in Low and High IPS Groups of Advanced-stage classical Hodgkin Lymphoma. **A** Morphology of a case with low IPS (HE 400×). **B** Expression of CTLA-4 in the low IPS case (IHC 400×). **C** Case with high IPS (HE 400×). **D** Expression of CTLA-4 in the high IPS case (IHC 400×)

Evaluation of CTLA-4 expression was performed by researchers blinded to the clinical data. Lymphocytes and histiocytes that showed brown staining in the cell membrane or cytoplasm were considered positive. Photographs were taken of five well-stained high-power fields and the average number of positive cells per field was obtained and analysed (Fig. 1).

### Data analysis

All data were analysed using SPSS software version 25.0. Continuous variables are presented as the mean due to a normal data distribution using the Kolmogorov‒Smirnov test. The statistical analysis was performed using appropriate bivariate tests (unpaired T test). A p value of 0.05 was used as the threshold for statistical significance.

## Results

This study included 40 advanced-stage CHL cases involving 21 males (52.5%) and 19 females (47.5%). Age ranged from 12 to 70 years old: 35 (87.5%) of the patients were ≤ 45 years old. Nodular sclerosis (NS) was the predominant CHL subtype (62.5%), followed by mixed cellularity (MC) (27.5%), lymphocyte depleted (LD) (7.5%), and lymphocyte rich (LR) (2.5%). The tumor location were in the lymph nodes and extra nodal sites such as mediastinum, lung, and spleen. The predominant lymph nodes affected were the cervical region. The cases were divided into advanced-stage CHL with low IPS score and advanced-stage CHL with high IPS score. The high IPS score group showed lower Hb levels, lymphocyte counts, and albumin levels and higher leukocyte counts than the low IPS score group. It was notable that more advanced disease stages increased as the IPS increased, as highlighted by none at stage 2 and 80% at stage 4 in the high IPS group, in contrast to 15% at stage 2 and a lower number at stage 4 (45%) cases in the low IPS group. The demographic and clinicopathologic characteristics of each group in this study are presented in Table 1.

**Table 1:** Demographic and clinicopathologic characteristics of advanced-stage CHL patients

Variable	Advanced-stage CHL	
	Low IPS N = 20	High IPS N = 20
Age (years, mean ± SD)	32.55 ± 14.13	30.80 ± 13.63
Sex, n (%)		
Male	9 (45%)	12 (60%)
Female	11 (55%)	8 (40%)
Subtype, n (%)		
Nodular sclerosis	13 (65%)	12 (60%)
Mixed cellularity	5 (25%)	6 (30%)
Lymphocyte depleted	1 (5%)	2 (10%)
Lymphocyte rich	1 (5%)	0
Haemoglobin (g/dl, median (min–max))	11.6 (2.7–14.1)	9.5 (7.4–14.6)
Leukocyte (/µL, mean ± SD)	11.216 ± 5320.96	21.659.8 ± 15.310.17
Lymphocyte (%, median (min–max))	13.5 (6.1–41.4)	4.3 (1.3–12.2)
Albumin (g/dl, mean ± SD)	3.67 ± 0.53	2.75 ± 0.52
Stage, n (%)		
2	3 (15%)	0
3	8 (40%)	4 (20%)
4	9 (45%)	16 (80%)

IPS: International Prognostic Score; CHL: Hodgkin lymphoma; Min: minimum; Max: maximum; SD: standard deviation

**Fig. 2:**
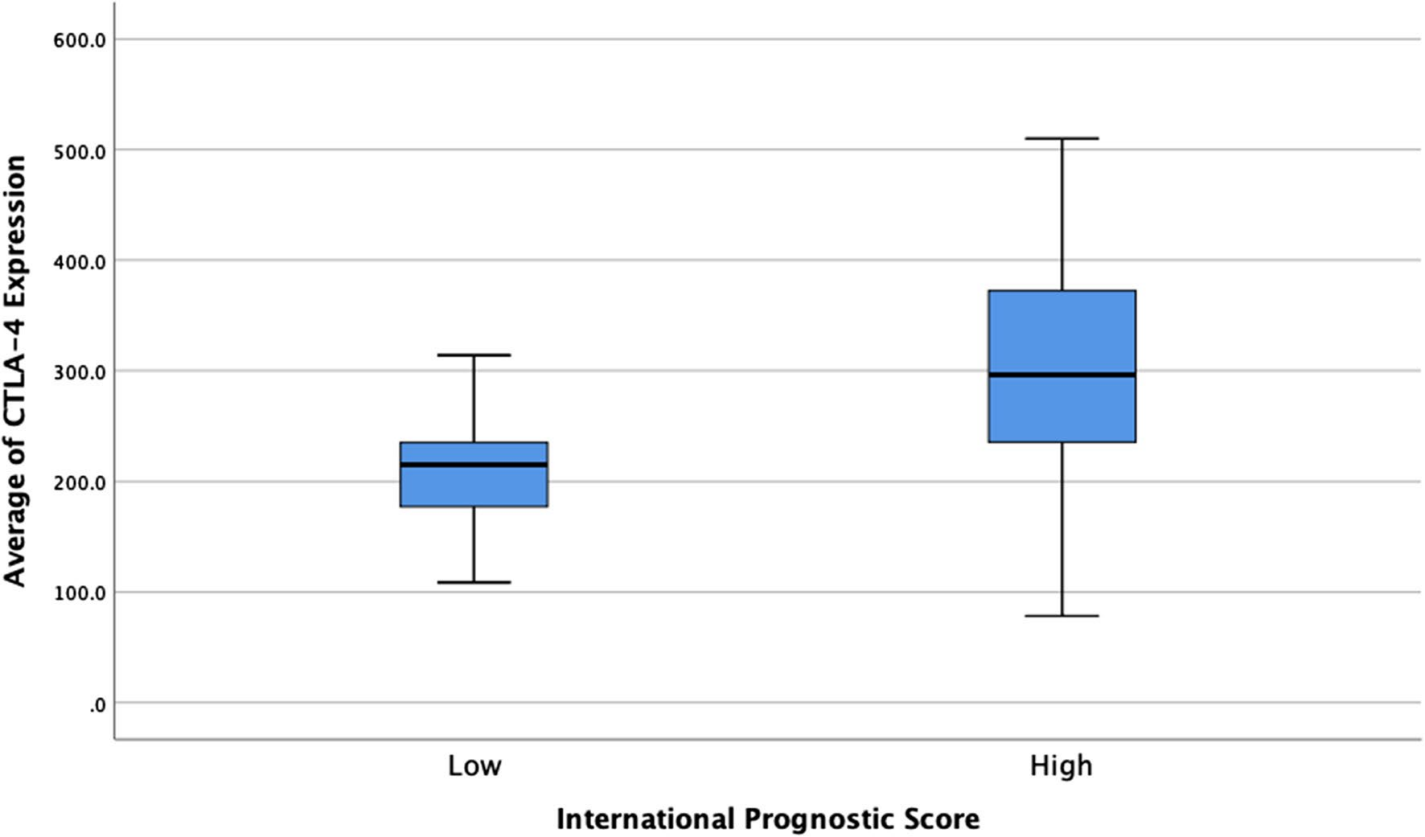
CTLA-4 expression in the low and high IPS group

Table 2 reveals that the average CTLA-4 expression in the ≤ 45 years old, female, Hb < 10.5 g/dl, leukocyte > 15,000/µL, lymphocyte < 8%, and albumin < 4 g/dl group was higher than that in the opposite group (Table 2). These cut-off points were determined according to the IPS criteria. Crucially, there was notably higher CTLA-4 expression in stage 4 disease than in disease less than stage 4, suggesting that CTLA-4 expression might correlate with more advanced disease. This result was supported by the fact that the advanced-stage CHL with high IPS group had a significantly higher average CTLA-4 expression than the low IPS group (p value 0.003) (Fig. 2).

**Table 2:** CTLA-4 expression in advanced-stage CHL and its relationship with IPS

Variable	CTLA-4 expression (mean ± SD)	p value
Age		
≤ 45 years old	258.59 ± 98.49	0.757
> 45 years old	243.64 ± 115.25	
Sex		
Female	271.2 ± 107.67	0.387
Male	243.62 ± 91.56	
Haemoglobin		
< 10.5 g/dl	285.85 ± 115.31	0.077
≥ 10.5 g/dl	230.36 ± 75.46	
Leukocyte		
≤ 15,000 µ/L	219.11 ± 74.31	0.004
> 15,000 µ/L	307.6 ± 107.79	
Lymphocyte		
< 8%	279.62 ± 122.07	0.107
≥ 8%	228.74 ± 51.68	
Albumin		
< 4 g/dl	261.06 ± 103.57	0.414
≥ 4 g/dl	217.65 ± 24.07	
Stage		
< 4	210.23 ± 84.94	0.020
4	284.62 ± 98.12	
IPS		
Low IPS	210.11 ± 52.41	0.003
High IPS	303.33 ± 113.52	

Unpaired T test; CTLA-4: cytotoxic T-lymphocyte-associated protein 4; SD: standard deviation

**Fig. 3:**
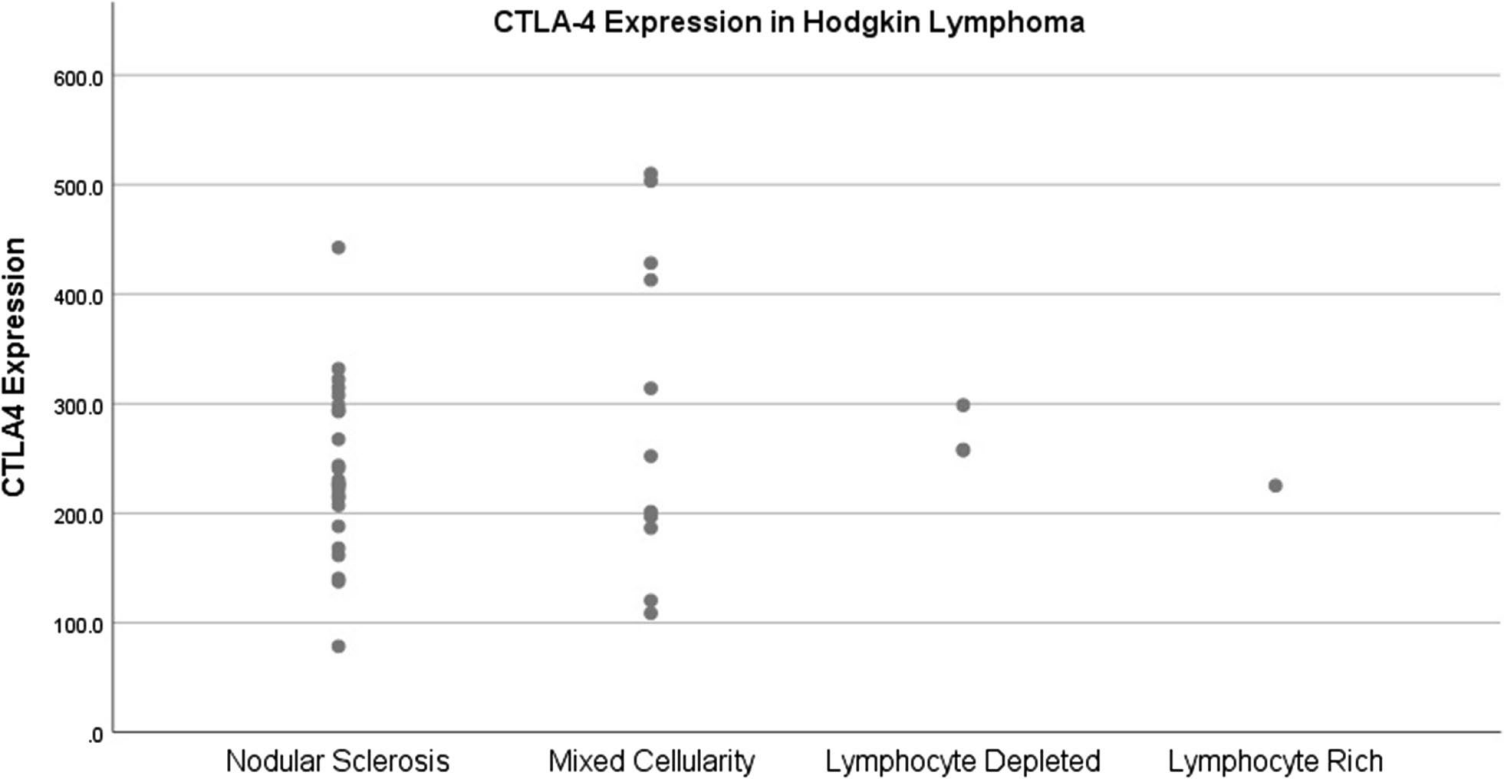
A scatter plot of CTLA-4 expression in CHL subtypes

A detailed breakdown of CTLA-4 expression in CHL subtypes is presented in Table 3. The findings revealed that CTLA-4 expression was notably highest in the MC subtype, followed by the LD, NS, and LR subtypes. A scatter plot of CHL subtypes displayed in Fig. 3.

**Table 3:** Average CTLA-4 expression in every CHL subtype

Variable	Average of CTLA-4 expression (mean ± SD)
CHL subtypes	
Nodular sclerosis	239.84 ± 76.36
Mixed cellularity	293.95 ± 147.94
Lymphocyte depleted	271.4 ± 23.56
Lymphocyte rich	225.2

CTLA-4: cytotoxic T-lymphocyte-associated protein 4

## Discussion

Although CHL generally has good prognosis, some CHL cases may be refractory to treatment and are usually diagnosed at an advanced stage and prone to recurrence. The IPS has a crucial role in CHL management by including several prognostic indicators to stratify and modify therapy and predict relapse or recurrence. Each adverse prognostic indicator that is present is given a point, and the total score correlates with the expected CHL outcome. Advanced-stage CHL with higher IPS will have poorer prognosis and higher risk of recurrence or relapse. In contrast, lower IPS indicates a more favourable prognosis; hence, each patient can be treated accordingly [6].

CTLA-4 is a regulatory molecule that inhibits T-cell activation. The correlation between IPS and CTLA-4 expression is important as one of the putative ICIs because it may shed light on the mechanism of immune evasion disease outcomes. It has been observed that CTLA-4 is expressed differentially in patients with variable IPS levels, which may be related to the disease's aggressiveness. There seems to be a correlation between higher CTLA-4 expression and higher IPS, which suggests that higher IPS CHL may have more severe immune suppression, indicating disease severity and progression [2–5, 7]. In this study, only leukocytes count and tumor stage that were significantly associated with CTLA-4 expression with p-values 0.004 and 0.020, respectively.

The MC and LD subtypes had the two highest average CTLA-4 expression, 293.9 ± 147.9 cells/FOV and 271.4 ± 23.6 cells/FOV, respectively, while the LR subtype had the lowest average CTLA-4 expression (225.2 cells/FOV). These variations may reflect the distinct histological composition and TME of each subtype. Some studies have reported that the MC and LD subtypes of CHL generally have worse prognosis than the NS and LR subtypes. This is because the MC and LD subtypes are more common in patients of older ages, males, and advanced-stage patients and are associated with HIV and EBV infection. The MC subtype showing the highest CTLA-4 expression presumably correlates with a more immunosuppressive component due to the higher diversity of TME components that may modify the immune response. In contrast, the LR subtype consists of a more homogenous lymphocyte background, suggesting a less suppressed immune response, scarce tumour cells, and better prognosis, as reflected by lower CTLA-4 expression. The NS and LR subtypes are more common in cases involving early stages, young age, and low tumour cell abundance [2, 15, 16].

This study found that extranodal lesions were more common in the high IPS group. The high IPS group also tended to have more stage 4 disease. In line with the increase in CHL stage, CTLA-4 expression was increased and marked more aggressive and extensive disease. This implies that immunological checkpoints such as CTLA-4 are upregulated when CHL progresses, potentially aiding tumour cells in immune evasion and enabling them to multiply and spread with reduced immune system interference [17–21]. In this study, stage 4 cases had significantly higher average CTLA-4 expression than cases with staging below 4, with 284.6 ± 98.1 cells/FOV and 210.2 ± 84.9 cells/FOV, respectively.

Advanced-stage CHL with low IPS was found to have significantly lower CTLA-4 expression than the high IPS group (210.1 ± 52.4 cells/FOV vs. 303.3 ± 113.5 cells/FOV). This finding is similar to the study by Chen Y et al. [22], in which there was a significant difference between the average optical density of CTLA-4 in high- and low-risk groups of DLBCL patients (p < 0.05). High IPS and increased expression of CTLA-4 in advanced-stage CHL align with the theory of immunosuppressive status in more aggressive disease states. As an ICI, CTLA-4 decreases the immune response, and its increased expression can help the tumour to modify the immune response, hence causing a greater disease burden [17–21].

In conclusion, our findings suggest that CTLA-4 expression correlates with the worst prognostic factors in the IPS for advanced-stage CHL, supporting the notion that immune checkpoints play a role in cancer progression. This result supports the potential use of ICI inhibitors in CHL patients with high CTLA-4 expression and adverse IPS factors as personalized treatment based on immunological profiles, Additionally, it may lead to integration of immunological markers into prognostic scoring systems for CHL in the future.

## Limitations

The study was limited by the small sample sizes across each subtype. It did not employ additional modalities such as flow cytometry or Western blot, nor did it include double staining of IHC markers for CTLA-4 and other markers of T lymphocytes, macrophages, and granulocytes. Additionally, the study had limited clinical data, such as survival outcomes. We also suggest that future studies use larger sample sizes to analyze subgroups based on histopathology and other factors that encompass the IPS.

## Data Availability

The datasets generated and/or analyzed during the current study are available from the corresponding author upon reasonable request.

## Abbreviations

Ccl-20: C–C Motif Chemokine Ligand 20
Ccl-5: C–C Motif Chemokine Ligand 5
CHL: Classical Hodgkin Lymphoma
CTLA-4: Cytotoxic T-Lymphocyte-Associated Protein 4
EBV: Epstein‒Barr virus
FFPE: Formalin-fixed paraffin block-embedded tissue
Hb: Haemoglobin (Hb)
HIV: Human immunodeficiency virus
ICIs: Immune checkpoint inhibitors
IHC: Comprehensive Immunohistochemistry
Il-10: Interleukin 10
Il-6: Interleukin 6
IPS: International Prognostic Score
LD: Lymphocytes depleted
LR: Lymphocyte rich
MC: Mixed cellularity
NLPHL: Nodular lymphocytic predominant Hodgkin lymphoma
NS: Nodular sclerosis
Tgf-Β: Transforming Growth Factor Beta
TME: Tumour microenvironment
Tnf-Α: Tumour Necrosis Factor Alpha
